# Functions of the Brain

**Published:** 1855-09

**Authors:** 


### EDITORIAL DEPARTMENT.

*Functions of the Brain.*—Among the original communications in the July number of the "British and Foreign," we find one entitled "Further Researches into the Functions of the Brain. By Thomas Laycock, M. D."

Dr. Laycock is a man long distinguished for his scholarship in this department of physiology, and is widely known as the translator of "Unger and Prochaska on the Nervous System," one of the publications of the Sydenham Society. Dr. Laycock is a warm admirer of Unzer, and that with no little reason. Unzer's principal work was published in 1771, at a period when the Stahlian philosophy (under which he was educated) was in vogue, and when a true physiology, based on experiment and anatomy, was hardly known. Valsalva, Spallanzani, Haller, Hoffman, Willis and a few others, should indeed be excepted from this statement, but even to those most familiar with the philosophical spirit that animated these men, it is a matter of no small wonder that Unzer should have promulgated doctrines so far in advance of his age; doctrines which only acquire new force in the progress of experimentation, and which even yet remain unexhausted. A quotation or two from Unzer would show, had we space, that not only was he in advance of Marshall Hall in the appreciation of reflex action, so far as the spinal cord is concerned, but that he took a deeper view of the functions of the cerebrum, and was the first to fully appreciate that class of phenomena now known under the name of "involuntary cerebration," or reflex actions of the brain proper.

We have taken occasion thus to mention Unzer, from the fact that his doctrines seem to have greatly influenced Dr. Laycock, whose reasoning runs parallel with Unzer's theory.

Dr. Laycock, in the article under notice, handles the subject of cerebral reflex action in the most forcible and logical manner. It will be recollected that Marshall Hall in a manner divides from each other the brain, the spinal cord, and the ganglionic system, assigning to the first the intellectual actions and the harmonizing muscular movements; to the second, the control of reflex actions, and to the last the guidance of organic or involuntary life. Thus we have three distinct nervous systems, each in a manner independent of each other, and as a corollary, endowed with a special *vis nervosa* or mind.

Carpenter enlarges upon this. He recognizes the fact that many muscular movements originating in the brain, are as purely reflex or instinctive as those attributed to the spinal cord. Associated with this, also, is the identity of structure of the brain and spinal cord, and the unity of their ganglionic arrangement.

It is some months since we have looked at his pages; but, if we recollect Carpenter aright, he traces the phenomena of motion and thought in this wise:

An impression is produced upon the periphery of a nerve; this conveyed to the cord results in a sensation and the sensation in a motion — always conservative and instinctive, and accomplished by the forces of the cord alone, without implicating the consciousness.

Should the brain, however, be called into action, the sensation results in an entirely distinct phenomenon — an idea — the perception causes an emotion, the emotion an intellectual operation, which finally calls into action the will.

It is Dr. Laycock's object to prove that, not only is the brain itself the recipient of impressions and the subject of sensations without any result further than unconscious reflex action, but that perception and emotion are themselves reflex actions, not necessarily implying consciousness, but entirely dependent on that conservative nervous force, which, beginning with the first cell of the embryo man, watches over and repairs his body till the final withdrawal of life.

It is upon this point that Dr. L. differs from Dr. Carpenter, who asserts that there is an essential distinction, anatomical and physiological, between the *sensory* and the hemispheric ganglia — that is, the laws of reflex action do not apply to the latter.

What is reflex action? It is the intelligent but unconscious respondence to stimuli. Under this broad definition it will be seen at once that reflex action is common to all forms of nervous arrangement, and not only to animal tissues, but to vegetables. Many of these latter manifest in the fullest degree that unconscious intelligence which guards from injury, shrinking from the touch, or opening in one case, closing in another, to the sunlight. It is proper to call this force an intelligence, inasmuch as it acts uniformly with regard to the good of its possessor. It is the Archæus, the *vis medicatrix*. It is, according to Dr. L., an endowment of the primitive cell, beginning with the first contact of the sperm-cell and germ-cell, a power which governs all additions to that cell up to the period of full grow.h, and after that guides the repair and waste of tissues, and is still identical with the power of reflex action.

Another feature of the intelligence of reflex action, is its *inspiration*, the uniform accuracy of its movements, and the wonderful knowledge it displays of the laws of nature. The tree turns its leaves to the sun to realize the same change wrought by the photographist upon sensitive paper, the spider spins its web with the most scientific adaptation to the laws of tension; the bee constructs its cell with such reference to geometrical laws, that, according to Sydney Smith, "it would take a senior wrangler at Cambridge, ten hours a-day, for three years together, to know enough mathematics for the calculation of these problems, with which not only every queen bee, but every undergraduate grub, is acquainted when it is born." Here is the wonder—with the bee it is not example or education, it is intuition.

It is a singular reflection that as the hemispherical ganglia increase in size, as the cerebrum becomes more and more the organ of the will, and subject to the control of reason in the increasing scale of creation, that this *constructive* faculty is lost. As we come to use tools, the capacity for instinctive architecture is diminished.

This instinctive use of the apparatus used by the unconscious mind (*vis nervosa*) is usually considered as distinct from the instructive construction of new or more fitting apparatus. Aside from the conclusions readily drawn from foregoing facts, it is evident that the adaptation of the form of plants or animals to new conditions or circumstances, and their ready return to their original form on the withdrawal of those conditions, is a fact going far to prove the identity of the unconscious mind with the reparative force, or *vis medicatrix*. Analogous to this is the fact that natural instincts, though suppressed, are never eradicated. The horse, who for many generations has known no danger from wild beasts, still preserves his instinctive fear of them

Perhaps the most striking train of ideas supported by Dr. Laycock's article, is that which supposes immense, and, perhaps, perfect knowledge of all natural science on the part of the unconscious mind. It is seen that this force always acts in accordance with the dictates of philosophy, that its creations combine—always—beauty with utility, that it confers upon the bee the benefit of exact knowledge of geometrical rules, endows the insect with all that foresight necessary for reproduction and provision for the necessaries of its young, and confers upon the unconscious, unreasoning, and unexperienced infant all the powers necessary to its sustenance, while it imparts to the mother the sacred and inviolable impulse to cherish it, an impulse wisely removed from the action of the intellect; for who can doubt that were reason alone to settle the question, few mothers would preserve their young?

The existence of these and a thousand similar unconscious powers—innate and inseparable from nature—implies the existence of an unconscious reason, removed from our perceptions. Such an existence also implies mind, knowledge, foresight, precaution, fear, pleasure, all the phenomena of the conscious mind except perception, ideas and memory.

And what a knowledge must this be which builds the human form upon a model strictly geometrical as to strength, purely æsthetic as to beauty, which balances it in locomotion with the nicest appreciation of the laws of gravity, which constructs the tissues here firm, there soft, with closest regard to resistance, tension, and compression; which surpasses the optician in adaptation to the laws of light, or the engineer in hydrostatics, and which in its marvellous laboratory works out the most difficult analyses, or combines the most unwilling elements, with an accuracy unattainable by rational chemistry.

These are the *substrata* of mind, the source of knowledge. In this sense nothing can be said to transcend the human mind. Could the mine of the unconscious mind be developed, every problem of science would be capable of solution at a glance; in the twinkling of an eye, and without resort to that succession of ideas which we call reason. We should solve our present enigmas as the natural arithmetician adds, subtracts, divides, or multiplies the largest masses of figures, in a moment, with unerring certainty, but without knowing how he does it.

Let once the connection between the conscious mind be perfected, conveying instantaneously to the former the conceptions of the latter, and books would be useless, the superiority of intellect would be gone, and universal equality competent to seize the advantages offered them by nature, and profit by them.

The present inequality in the powers of races and individuals would seem in this light to depend upon two causes:

1st. The differences in the perfection of the material organism in its size, shape, and molecular construction.

2d. The differences in the degree of intercourse and relationship between the unconscious and the conscious mind, that is, in the capacity of the latter to appreciate and use the intuitions of the former.

In the first class the abscence or malformation of the hemispherical ganglia, as in acephalous infants, leaves the subject of it entirely to the instinctive faculties. The child breathes, suckles, swallows, starts at noise, or shrinks from pain, like any other; but it is utterly shut out from communication with the outer world, and can never appreciate the powers which it momently applies. Not far different are those cases where, from some error in the molecular arrangement of the brain, consequent upon scarlatina or other infantile disease, the once bright child sinks down to idiocy. Here only the instinctive powers remain, the uses of language or the hundred ways in which the ordinary child takes advantage of its innate knowledge of natural science are wanting, and lead us to regret that the idiot could not share in the early death allotted to the acephalous child.

This is only looking at a well known phase of life from a new stand-point, it involves no new theory, and is the simple enunciation of the axiom that the functional power of an organ depends upon its physical integrity.

The other proposition involves the idea of cerebral activity. Aside from those powers of mind due to "acquired knowledge," it implies an intrinsic fruitfulness of the brain, such as is usually designated "genius" or "talent." But as it is evident that the law of healthy exercise applies as well to the brain as to other organs, it follows that the cerebral organ cannot be in an advantageous position for realizing the benefit of the knowledge of the unconscious mind, without it is itself in an active and healthy condition. The stimulus of this health is acquired knowledge, and though genius or talent may in themselves occasionally develop results, it is evident that without acquired knowledge to call it forth, intuitive knowledge—genius—must, in most cases, lie dormant.

Dr. Laycock, then, (for it should be recollected that we are not writing our own but condensing Dr. L.'s opinions) acknowledges the existence of genius as a distinct power of the mind, which he would probably define as a capacity on the part of the reason to profit by the intuitions of the unconscious mind.

There is, at least, comfort for the blockheads in this theory. The unconscious mind of the idiot may be as great a magazine of knowledge as that of Newton, but like the starling, "it can't get out"—it has no tools to work with.

